# Epigenetics Underpinning the Regulation of the CXC (ELR^+^) Chemokines in Non-Small Cell Lung Cancer

**DOI:** 10.1371/journal.pone.0014593

**Published:** 2011-01-27

**Authors:** Anne-Marie Baird, Steven G. Gray, Kenneth J. O'Byrne

**Affiliations:** 1 Thoracic Oncology Research Group, Institute of Molecular Medicine, Trinity College, Dublin, Ireland; 2 HOPE Directorate, St. James's Hospital, Dublin, Ireland; The University of Hong Kong, China

## Abstract

**Background:**

Angiogenesis may play a role in the pathogenesis of Non-Small Cell Lung cancer (NSCLC). The CXC (ELR^+^) chemokine family are powerful promoters of the angiogenic response.

**Methods:**

The expression of the CXC (ELR^+^) family members (*CXCL1-3/GROα-γ*, *CXCL8/IL-8*, *CXCR1/2*) was examined in a series of resected fresh frozen NSCLC tumours. Additionally, the expression and epigenetic regulation of these chemokines was examined in normal bronchial epithelial and NSCLC cell lines.

**Results:**

Overall, expression of the chemokine ligands (*CXCL1, 2*, *8*) and their receptors (*CXCR1/2*) were down regulated in tumour samples compared with normal, with the exception of *CXCL3*. *CXCL8* and *CXCR1/2* were found to be epigenetically regulated by histone post-translational modifications. Recombinant CXCL8 did not stimulate cell growth in either a normal bronchial epithelial or a squamous carcinoma cell line (SKMES-1). However, an increase was observed at 72 hours post treatment in an adenocarcinoma cell line.

**Conclusions:**

CXC (ELR^+^) chemokines are dysregulated in NSCLC. The balance of these chemokines may be critical in the tumour microenvironment and requires further elucidation. It remains to be seen if epigenetic targeting of these pathways is a viable therapeutic option in lung cancer treatment.

## Introduction

Angiogenesis is important in the growth and spread of cancer and also influences inflammatory changes which may pre-dispose to the disease [1]. The CXC (ELR^+^) chemokines induce angiogenesis and may be important in cancers that have an angiogenic phenotype such as NSCLC [2].

The term chemokine refers to a family of low molecular weight (8–10 kDa) chemotactic cytokines. Chemokines are small inducible cytokines, which are chemo-attractants for leukocytes. Chemokines are classified by their amino acid composition, functional activity and receptor binding properties and comprise of four sub families defined according to the first two of four conserved cysteine residues (a) C, (b) CC, (c) CXC and (d) CXXXC [3]. The CXC chemokine family consists of two subtypes, ELR^+^ and ELR^−^, according to a particular Glu-Leu-Arg (ELR) motif preceding the first cysteine residue [3]. CXC (ELR^+^) promoters contain a putative *cis* element that recognises NF-κB, and therefore can cause the trans-activation of CXC chemokines [4].

The angiogenic receptor for CXCL8 and the other CXC (ELR^+^) chemokines is CXCR2 [5]. Blockade of this receptor leads to a decrease in angiogenesis in pancreatic cancer [6], and a significant inhibition of human melanoma tumour growth and experimental lung metastases in CXCR2−/− mice, as well as a reduction in angiogenesis [7]. Within the setting of the lung, cancer growth and metastatic potential is down-regulated in several mouse CXCR2−/− models [7], [8]. However, CXCL8 can bind to CXCR1 and CXCL1/CXCL8 can also bind DARC, although binding to DARC does not transduce a signal. Currently, studies with DARC suggest that it acts by ‘mopping’ up chemokines and, therefore, reducing their signalling capacity. Over-expression of DARC leads to increased tumour growth, however, this was due to the induction of large necrotic areas within the tumour [9], [10].

Chemokine receptors are up-regulated on tumour cells, allowing the tumour to take advantage of chemokine rich environments, promoting tumour growth and vasculature. In addition, chemokines can recruit macrophages, which detect the hypoxic environment within the tumour and subsequently secrete pro-angiogenic factors [11], [12]. Initially chemokines were thought to only play a role in attracting specific leucocytes to a site of injury; nevertheless it has now been shown that they are involved in the neoplastic transformation of a cell, promotion of angiogenesis, tumour clonal expansion and changes in the ECM, and in particular mediate organ specific metastases in cancer [13]. Specific ligand receptor pairs dictate the metastases patterns of breast and lung cancer [14]. In breast cancer metastases to the lung, CXCL1 was part of a gene signature that also included VCAM1 and MMP1 [15]. A recent study found that tumour derived CXCL8 acted as an attractant for circulating tumour cells to return to the original tumour, leading to a more aggressive tumour phenotype [16].

A variety of CXC chemokines have been detected in neoplastic tissues as products of tumour cells or stromal elements [12]. For example, tumour infiltrating inflammatory cells elevates CXCL8 levels in bronchioalveolar cells, along with its two receptors [17]. Strong evidence suggests that CXC (ELR^+^) chemokines have a role in cancer promotion, as they can promote growth and survival of cancer cells [18].

The growth and progression of cancer is dependant on angiogenesis and CXCL8 has been demonstrated to play a role in its angiogenic and tumourigenic potential. In renal cell cancer the levels of CXCL1, CXCL3 and CXCL8 were elevated compared to controls and in receptor negative (CXCR2−/−) mice there was a corresponding reduction in tumour growth [19]. Studies using melanoma tumour models support the role of CXCL1, CXCL2, and CXCL3 in mediating tumour angiogenesis and levels of all three chemokines are highly expressed in melanoma tumours. Transfection of CXCL1–3 into immortalised non-tumorigenic cells gave them the ability to form tumours [20], [21]. CXCL8 is one of the most studied members of the CXC (ELR^+^) family, particularly in lung cancer. CXCL8 was identified in a gene expression signature that was predicative of poor prognosis in patients with stage I lung cancer [22], while levels of CXCL8 are significantly increased in both malignant pleural effusions [23] and NSCLC [24], where levels increase with stage [25] and correlate with patient survival/relapse [26].

Chemokines found within the tumour microenvironment are thought to play at least five roles in the development of tumours and metastatic disease; (a) control leukocyte infiltrate, (b) modify tumour immune response, (c) regulate angiogenesis, (d) operate as growth and survival factors, and (e) direct the movement of tumour cells themselves [27]. The CXC (ELR^+^) ligands and receptors are particularly important in mediating NSCLC tumour associated angiogenesis [28] and organ specific metastases [13], [14]. CXCR2 ligands have also been implicated in NSCLC tumour progression through Snail, high levels of which correlate with decreased survival [29].

Aberrant epigenetic regulation of gene expression is a frequent event in NSCLC [30], [31]. Targeting these epigenetic regulatory mechanisms in NSCLC is an active area of pharmaceutical research. We sought to examine the expression of the CXC (ELR^+^) chemokines and their receptors in a series of primary NSCLC tumour samples and in an additional panel of NSCLC cell lines, and to directly examine whether epigenetics plays a role in the regulation of these genes in this disease. Our results indicate that the expression of these chemokines and their receptors are frequently deregulated in NSCLC, being regulated via directly and in directly by epigenetic mechanisms (histone post-translational modifications and DNA CpG methylation, respectively) and may be good candidate targets for epigenetic therapy in the treatment of this cancer.

## Methods

### Cell lines

The A549 (adenocarcinoma), SKMES-1 (squamous cell carcinoma), H460, H647 and H1299 (large cell carcinoma) and BEAS-2B (transformed normal bronchoepithelial) cell lines were purchased from the ATCC (LGC Promochem, Teddington, UK). HBEC cell lines [32] were a gift from Prof. John D Minna (Hamon Centre for Therapeutic Oncology Research, UTSoutwestern, Dallas, TX, USA). All cell culture reagents were purchased from Lonza (Walkersville, MD, USA). Cells were maintained at 37°C in a humidified atmosphere containing 5% CO_2_ in the following media; A549 - F-12 (Ham) medium supplemented with 10% (v/v) FBS, penicillin streptomycin (500 U/mL) and 2 mM L-glutamine. BEAS-2B were also maintained in F-12 (Ham) medium without the addition of FBS. SKMES-1 - EMEM with the addition of 10% (v/v) FBS, penicillin streptomycin (500 U/mL), 2 mM L-glutamine and 0.1 M non-essential amino acids. All large cell carcinoma lines were maintained in RPMI with 10% FBS and penicillin streptomycin (500 U/mL). HBEC lines were maintained in Keratinocyte serum-free media (SFM), with L-glutamine (GIBCO Invitrogen, Paisley, Scotland) and supplemented with 2.5 µg human recombinant epidermal growth factor (rEGF), and 25 µg bovine pituitary extract (GIBCO Invitrogen).

### Primary tumour samples

A series of 37 tumour specimens (14 adenocarcinoma and 23 squamous cell carcinoma) were taken from patients presenting with early stage NSCLC at St. James's Hospital, Dublin. Matched normal tissue was taken in parallel for each patient and samples were evaluated by a pathologist immediately following dissection.

### Reagents

Trichostatin A (TSA) was purchased from Calbiochem (San Diego, CA, USA) and dissolved in DMSO to a concentration of 250 mg/mL. Cell cultures were treated for a period of 16 h, at a final concentration of 250 ng/mL.

Phenylbutyrate (PB) (Tributyrate™) was a gift from Triple Crown America, Perkasie, PA, USA. Cell cultures were treated at a final concentration of 10 mM for 16 h.

5-Aza-2′-Deoxycytidine (DAC) was purchased from Merck (Darmstadt, Germany) and dissolved in methanol. Cell cultures were treated with DAC (final concentration - 1 µM) for 48 h with DAC and media replaced every 24 h.

Recombinant human CXCL8 was purchased from PromoKine (PromoCell GmbH, Heidelberg, Germany) and reconstituted in sterile distilled water.

SB 225002 was purchased from Cayman Chemical (Ann Arbor, MI, USA) and dissolved in DMSO to a concentration of 2.2 µM. Cell lines were treated for one hour prior to the addition of CXCL8, at a concentration of 0.022 µM.

### Total RNA isolation and RT-PCR amplification

Total RNA was extracted using TRI reagent® (Molecular Research Center, Montgomery Road, OH, USA) according to manufacturer's instructions. Prior to first strand cDNA synthesis, 10 µg of total RNA was pre-treated by digestion with RQ1 DNase (Promega, Madison, WI, USA) according to the manufacturer's instructions. cDNA was generated using Superscript III (Invitrogen Corp., Carlsbad, CA, USA) and Oligo dT(20) primers (Eurofins MWG Operon, Ebersberg, Germany) according to the manufacturer's instructions.

Cell lines were examined for the expression of *CXCL1–3*, *CXCL8*, *CXCR1/2* and *Beta-actin* by RT-PCR, using primers and annealing temperatures outlined in Table 1. PCR cycling conditions consisted of −95°C for 5 min followed by 35 cycles of 1 min at 94°C, 1 min at the target gene annealing temperature and 1 min at 72°C with a final extension at 72°C for 10 min.

**Table 1:** Primers and annealing temperatures for RT-PCRs.

Primer Set	Sequence	Annealing Temp (°C)
CXCL1 (324 bp)	F: 5′-ATGGCCCGCGCTGCTCTCTC-3′	55
	R: 5′-TCAGTTGGATTTGTCACTGTTC-3′	
CXCL2 (324 bp)	F: 5′-ATGGCCCGCGCCACGCTCTC-3′	55
	R: 5′-TCAGTTGGATTTGCCATTTTTCAGC-3′	
CXCL3 (324 bp)	F: 5′-ATGGCCCACGCCACGCTCTCCG-3′	60
	R: 5′-TCAGTTGGTGCTCCCCTTGTTC-3′	
CXCL8 (297 bp)	F: 5′-ATGACTTCCAAGCTGGCCGTG-3′	55
	R: 5′-TGAATTCTCAAGCCCTCTTCA-3′	
CXCR1 (512 bp)	F: 5′-CCTTCTTCCTTTTCCGCCAG-3′	60
	R: 5′-AAGTGTAGGAGGTAACACGATG-3′	
CXCR2 (300 bp)	F: 5′-CTGCTCTGCTGGCTGCCCTA-3′	60
	R: 5′-GAGAGTAGTGGAATTGTGCCC-3′	
Beta-actin (510 bp)	F: 5′-TGTTTGAGACCTTCAACACCC-3″	55
	R: 5′-AGCACTGTGTTGGCGTACAG-3′	

Experiments were carried out in triplicate and products electrophoresed on a 1% agarose gel. Product quantification was performed using TINA 2.09c (Raytest, Isotopenmeßgeräte GmbH, Straubenhardt, Germany) densitometry software. The mRNA expression was normalised to *Beta-actin* controls, and was expressed as a ratio of target mRNA expression: *Beta-actin* expression.

### Chromatin immunoprecipitation (X-ChIP)

Chromatin immunoprecipitation was performed as follows: Following treatments, cells were fixed with formaldehyde (final concentration 1%), suspended in SDS lysis buffer (Millipore, Billerica, MA, USA) and sonicated until DNA was fragmented into lengths of between 200–1000 bp. Aliquots of this sheared DNA were subsequently immunoprecipitated using the OneDay ChIP Kit™ (Diagenode, Liege, Belgium) according to the manufacturer's instructions. The antibodies used for immunoprecipitation were as follows: pan acetyl-histone H3 (Millipore Cat#06-599), pan acetyl histone H4 (Millipore Cat# 06-598), acetyl-histone H3 (K9/14ac) (Diagenode Cat# pAb-ACHBHS-044), acetyl-histone H3 (K9ac) (Diagenode Cat# pAb-ACHAHS-044), acetyl phospho - histone H3 (K9pS10) (Sigma Cat# H0788), di methyl-histone H3 (K9Me2)(Sigma Cat# D5567), di methyl histone H3 (K4Me2) (Sigma Cat# D5692) and methyl-histone H3(K4Me) (Sigma Cat# M4819). A no antibody control was included to test for non specific binding.

Primers used to study the promoter regions of *CXCL8* and *CXCR1/2* by ChIP were designed from the known 5′ UTRs contained within their nucleotide sequences. RT-PCR cycling conditions were the same as those outlined above. Primers and annealing temperatures for ChIP are presented in Table 2.

**Table 2:** Primers and annealing temperatures for X-ChIP.

Primer Set	Sequence	Annealing Temp (°C)
CXCL8 (171 bp)	F: 5′-AAGAAAACTTTCGTCATACTCCG-3′	55
	R:5′-TGGCTTTTTATATCATCACCCTAC-3′	
CXCR1 (120 bp)	F: 5′-TCAACCCCAGCTTCACACCT-3′	56
	R: 5′-CCCCACCCATGTCTACATTT-3′	
CXCR2 (215 bp)	F: 5′-CCACTGGATCCCTTGCAGAC-3′	60
	R: 5′-GGAAACTTCCCACCAGTGAA-3′	

### ELISA

The concentration of CXCL8 was measured in conditioned media using a DuoSet® ELISA Development Systems (R & D systems, Minneapolis, MN, USA) in line with manufacturer's instructions, with one exception; the substrate solution used was made in phosphate citrate buffer (containing citric acid and Disodium hydrogen orthophosphate dodecahydrate, pH 5.0), 10 mg of 1, 2-phenylenediamine dihydrochloride (OPD) and 18 µL of 1 M H_2_O_2_.

CXCL2 was quantified using an ELISA development kit purchased from Strathmann Biotec (Hamburg, Germany), according to manufacturer's instructions.

### Cellular proliferation assays

Cell proliferation was measured using a Cell Proliferation ELISA, BrdU (Roche Diagnostics Ltd., Sussex, UK). Briefly, cells were seeded at 5×10^3^/well in a 96-well plate and adhered overnight. Subsequently the complete media was removed and the cells washed with 100 µL PBS. Serum depleted media (0.5% FBS) was added to the lung cancer cells only, as this mimics more closely physiological conditions. Following overnight incubation, cells were treated for 24, 48 or 72 h with human recombinant CXCL8 at various concentrations (0.1–100 ng/mL). Inhibition studies were carried out by pre-treating cells with 0.022 µM SB225002 for 1 h prior to the addition of CXCL8. Absorbance was measured on a plate reader at 450 nm with a reference wavelength set to 690 nm and blank and untreated wells were used for normalisation purposes. The untreated cells were set as 100%, and the CXCL8 treatments assessed relative to this.

### Statistical analysis

The data are expressed as mean ± SEM (standard error of the mean). Statistical analysis was performed with InStat (Graphpad software, La Jolla, CA, USA) using a paired one tailed Student's *t*-test. Differences were considered significant when p<0.05.

## Results

### Expression of *CXCL1–3*, *CXCL8* and *CXCR1/2* in primary lung cancer tumour specimens

**Figure 1:**
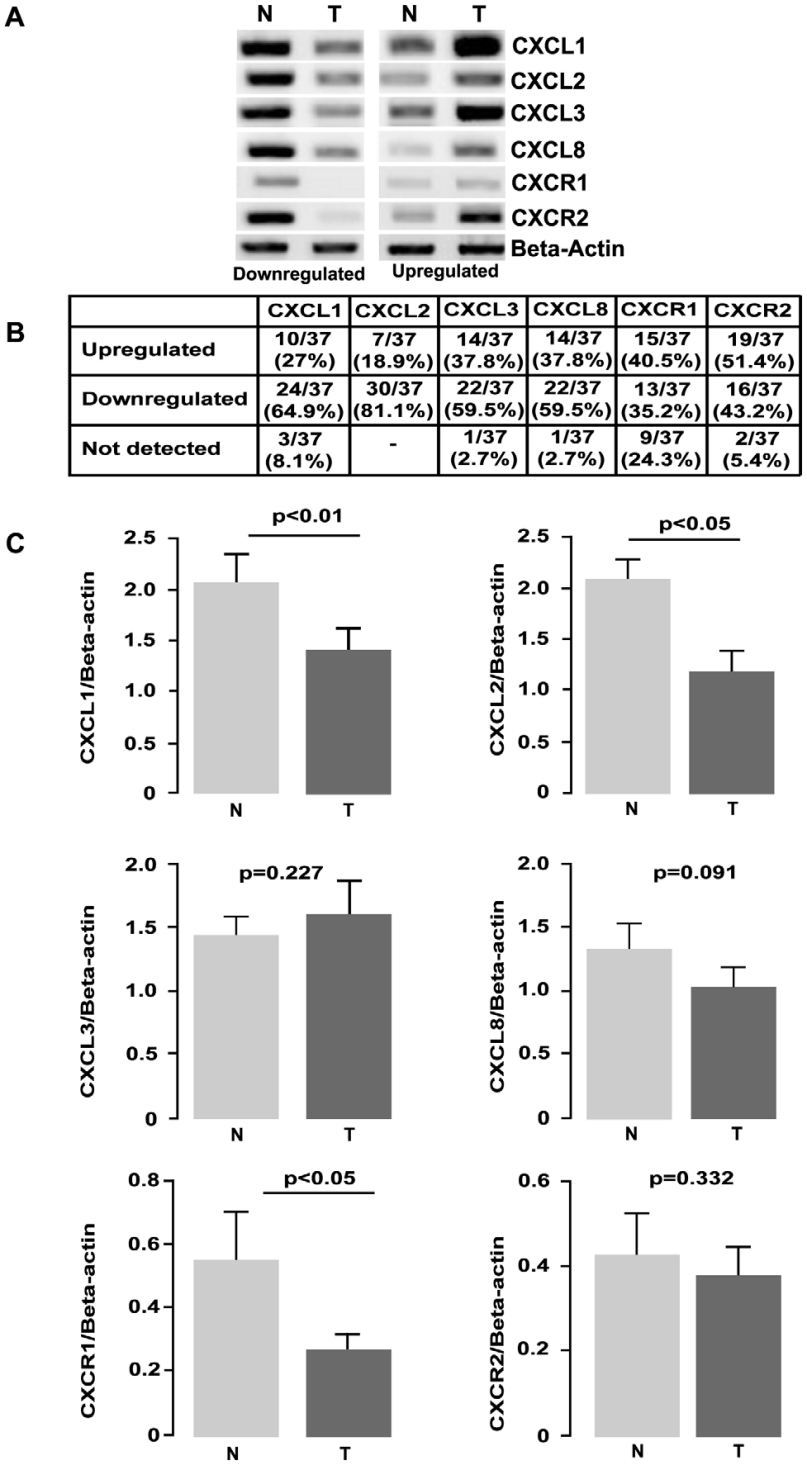
CXC (ELR^+^) chemokine family mRNA expression in normal/tumour NSCLC matched pairs. A) Levels of *CXCL1*–*3*, *CXCL8* and *CXCR1/2* were examined by RT-PCR on a panel of NSCLC lines (adenocarcinoma (n = 14), squamous cell carcinoma (n = 23)) patient samples. Representative images of up and down regulated samples are shown. *Beta actin* levels were used for normalisation purposes. B) A summary of the changes in expression of the various CXC (ELR^+^) chemokines and their receptors in a panel of tumour/normal matched patient NSCLC samples. C) Overall densitometry analyses of tumour/normal matched patient NSCLC samples. Data is graphed as mean ± standard error of mean (n = 37). (N – Normal, T – Tumour).

To assess the expression of a number of CXC (ELR^+^) family members in a panel of normal/tumour matched patient samples from Stage I and II patients, RT-PCR was performed (Figure 1A), and summarized in Figure 1B. Densitometric analysis of the RT-PCR revealed a significant decrease in the expression of *CXCL1*, *CXCL2* (p<0.01) and the *CXCR1* receptor (p<0.05) in NSCLC tumour samples compared with normal (Figure 1C).

### Expression of *CXCL1–3*, *CXCL8* and *CXCR1/2* in a panel of normal and lung cancer cell lines

**Figure 2:**
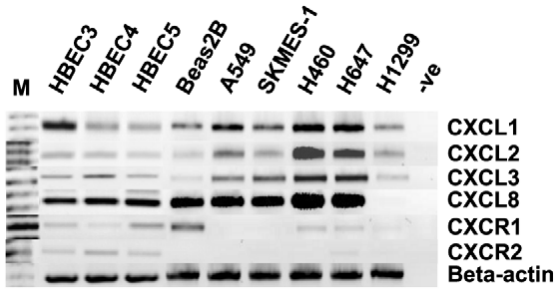
Expression of *CXCL1*–*3*, *CXCL8* and *CXCR1/2* in a panel of normal and NSCLC cancer cell lines. The panel included A549 (adenocarcinoma), SKMES-1 (squamous cell carcinoma), H460, H647 and H1299 (large cell carcinoma), BEAS-2B (SV40 transformed normal bronchoepithelial) and HBEC cell lines (normal bronchial epithelial cell lines immortalised in the absence of viral oncoproteins). *Beta actin* is included to validate loading efficiency. (M – DNA size marker, -ve – Negative RT-PCR control).

Utilizing RT-PCR, the chemokines were examined in a panel of normal and NSCLC cell lines (Figure 2). All cell lines tested expressed varying levels of *CXCL1–3* and *CXCL8*, with higher basal expression observed in the NSCLC lines. However, robust expression of both receptors (*CXCR1/2*) was detected in the normal cell lines (HBEC3–5).

### Histone acetylation is involved in the regulation of *CXCL8* and *CXCR1/2* expression

**Figure 3:**
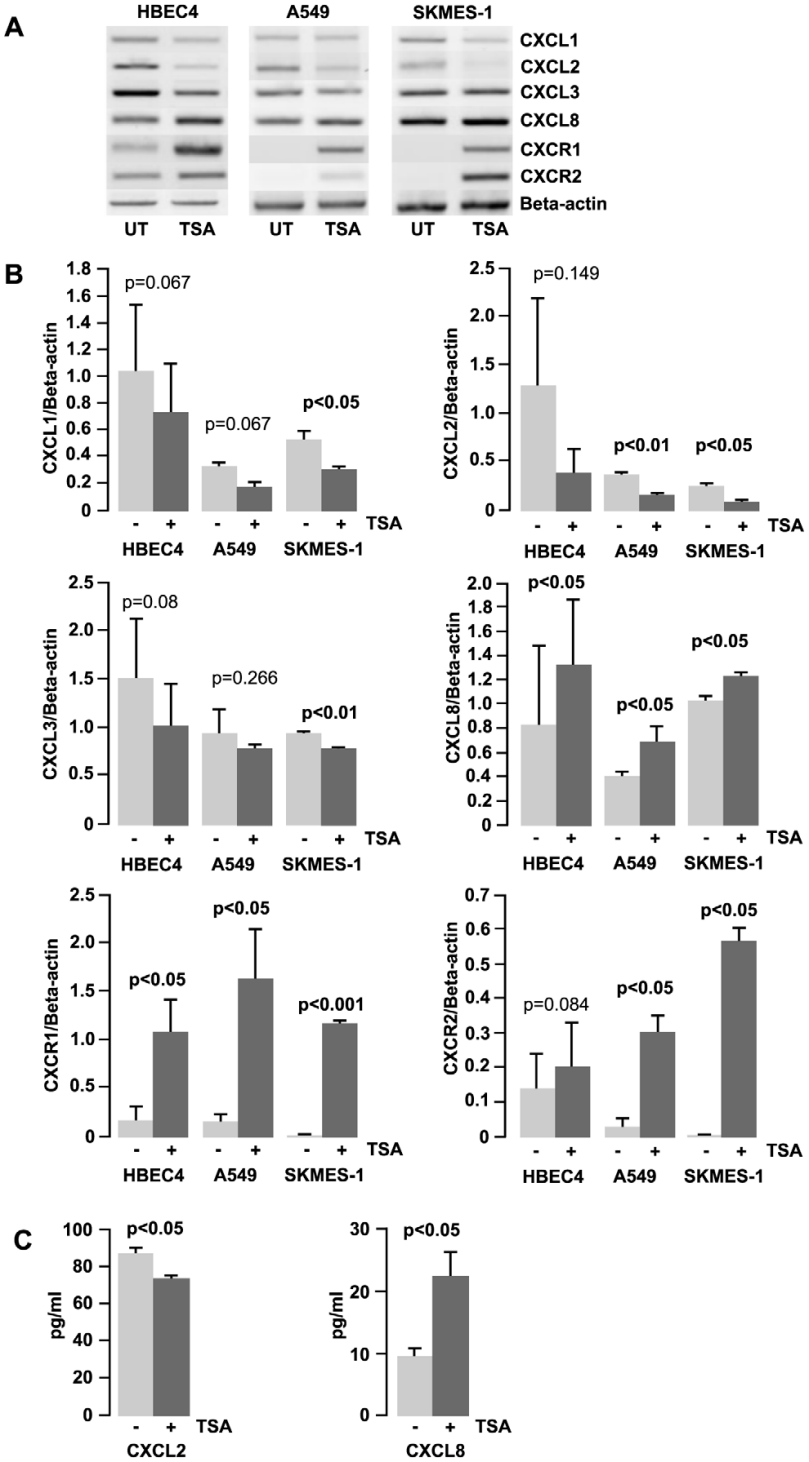
Cell line response to histone deacetylase inhibition. A) The effect of TSA treatment (250 ng/mL for 16 h) on the expression of *CXCL1*–*3*, *CXCL8* and *CXCR1/2*. B) Densitometry analysis of expression in treated versus untreated samples when normalised to *beta actin*. Data is graphed as mean ± standard error of mean (n = 3). C) Treatment with TSA also effects the production of CXCL2 and CXCL8 at protein level in SKMES-1 cells. Chemokines were quantified in conditioned media removed from culture after exposure to TSA (250 ng/mL for 16 h). Data is graphed as mean ± standard error of mean (n = 3). (UT – untreated, TSA – Trichostatin A).

Using the histone deacetylase inhibitor (HDACi), Trichostatin A (TSA), an induction of *CXCL8* and *CXCR1/2* in both the normal (HBEC4) and lung cancer cell lines (A549 and SKMES-1), with a concomitant decrease in *CXCL1–3* (SKMES-1, p<0.05) (Figure 3A) was observed. The induction of *CXCL8* was significant in HBEC4 and SKMES-1 (p<0.05), as was the increase in *CXCR1* and *CXCR2* in both the lung cancer cell lines (A549 - p<0.05, SKMES-1 – p<0.01) and *CXCR1* in HBEC4 (p<0.05) (Figure 3B). The reactivation of the expression of these receptors in the NSCLC cell lines would indicate that these genes are epigenetically regulated at the level of histone acetylation. Treatment of cell lines with an additional histone deacetylase inhibitor, phenylbutyrate (PB), also resulted in a reactivation of *CXCR1/2* (data not shown), indicating that the results are HDACi specific. Two chemokines were selected, CXCL2 and CXCL8, for the determination of protein expression by ELISA. The pattern observed reflected that seen at the mRNA level in SKMES-1, with a decrease in CXCL2 and an increase in CXCL8 with TSA treatment (p<0.05) (Figure 3C). However, there was no significant change in protein levels in either A549 or HBEC4 between basal and TSA treated cells (data not shown).

### Regulation of *CXCL8* and *CXCR1/2* occurs through direct chromatin remodelling

**Figure 4:**
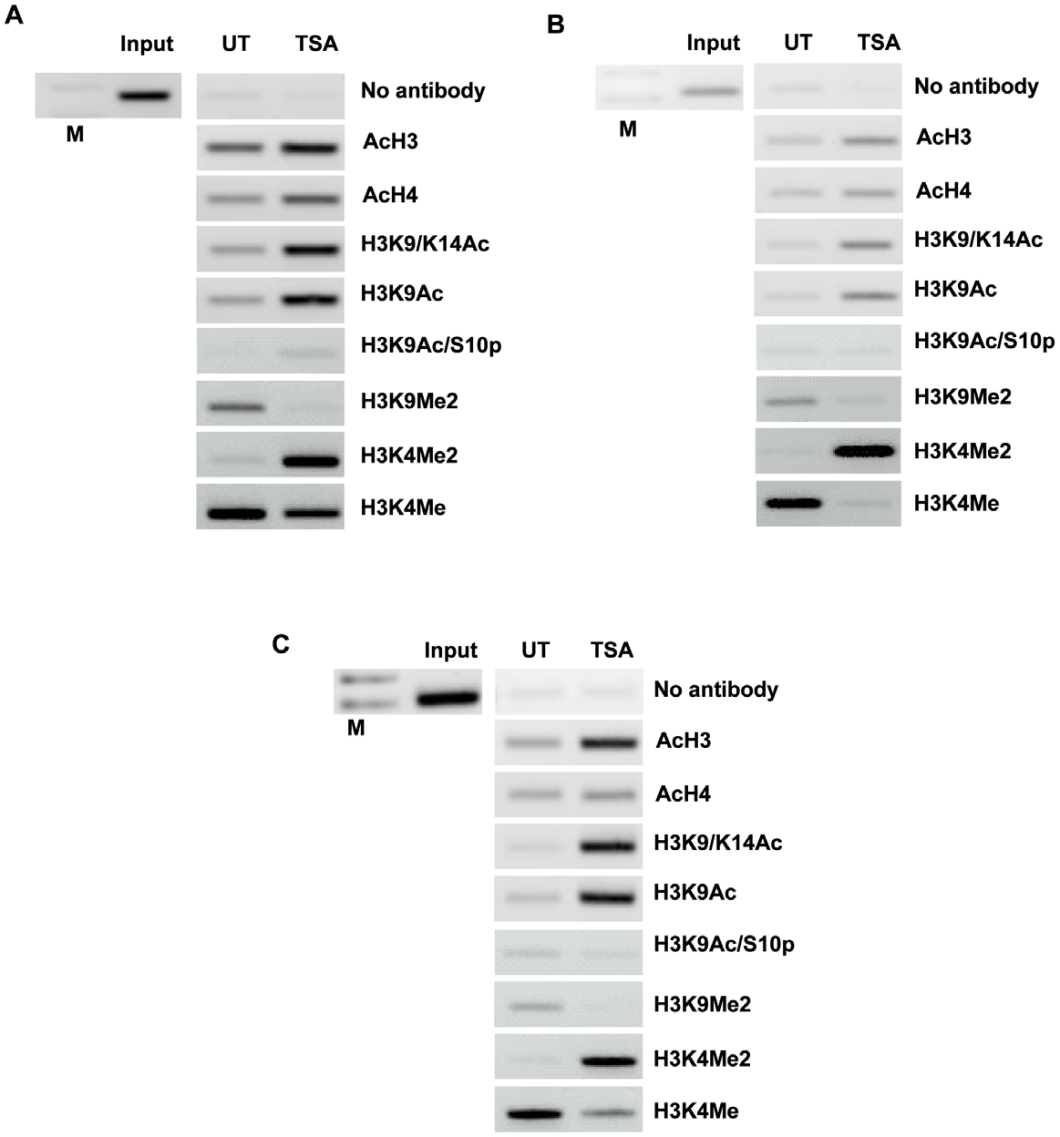
Histone acetylation occurs directly at the promoter regions of *CXCL8* and *CXCR1/2*. The ChIP assay demonstrates that TSA treatment results in an increase in the acetylation of histone H3 and H4. A549 cells were cultured in the presence or absence of TSA (250 ng/mL) for a period of 16 h. Subsequently, a ChIP assay was performed using the following antibodies; pan acetylated histone H3 (Ac H3) and H4 (Ac H4), histone H3 acetylated at lysine 9 and 14 (H3K9/K14Ac), histone H3 acetylated at lysine 9 (H3K9Ac), histone H3 acetylated at lysine 9 and phosphorylated at serine 10 (H3K9pS10), Histone H3 dimetylation marker at lysine 9 (H3K9Me2), dimetylation marker at lysine 4 (H3K4Me2) and methylation marker at lysine 4 (H3K4Me). The chromatin status at the promoter region of (A) *CXCL8*, (B) *CXCR1* and (C) *CXCR2* is shown. Input DNA serves as a positive control recommended by the manufacturer (Diagenode). A no antibody control was included to test for non specific carriage of DNA with histones. (M – DNA size ladder).

To confirm that the observed effects for HDACi were due to increased histone hyperacetylation at the promoters of the *CXCL8* and *CXCR1/2* genes, we carried out chromatin immunoprecipitation (ChIP) analysis of the individual promoters from A549 cells treated with TSA. As can be seen in Figure 4, treatment with TSA results in an increase in the amount of PCR product for *CXCL8* and *CXCR1/2* indicating enhanced histone hyperacetylation around the promoters for these genes. We show that lysine 9 and lysine 14 are hyperacetylated in this region following treatment with TSA. This experiment clearly demonstrates that chromatin remodelling is directly involved with the activation of *CXCL8* (Figure 4A), *CXCR1* (Figure 4B) and *CXCR2* (Figure 4C) gene expression. In addition, we also observed an increase in histone H3 lysine 4 tri-methylation (H3K4me3) at the *CXCR1* promoter (data not shown) and the phosphorylation of Serine 10 (H3K9pS10) at the *CXCL8* promoter. An increase in H3K4 diemethylation (H3K4Me2) was detected with a simultaneous decrease in H3K9 dimethylation (H3K9Me2) at the promoter region. These modifications have been associated with the activation of transcription and early response genes, respectively, and add additional strength to the evidence that these genes are dynamically regulated by histone pos-translational modifications.

### Methylation is not directly involved in the regulation of the CXC (ELR^+^) family

**Figure 5:**
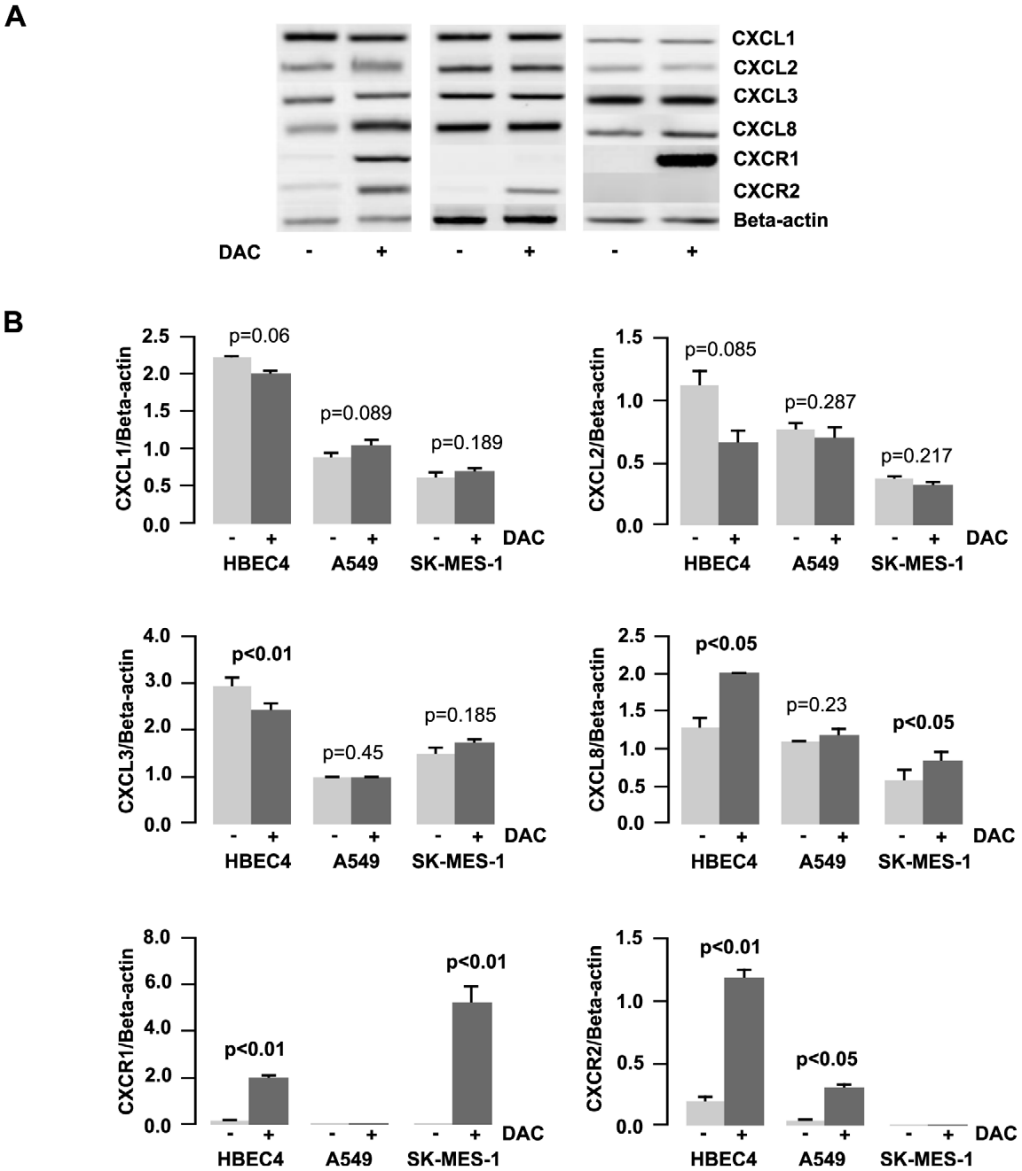
Cell line response to a DNA methyltransferase inhibitor. A) The effect of 5-aza-2′deoxycytidine (DAC) treatment on the expression of *CXCL1*–*3*, *CXCL8* and *CXCR1/2*. Cells were cultured in 1 µM DAC for 48 h with media and drug replaced every 24 h. Expression changes were measured using RT-PCR with Beta-Actin used as the internal control for quantification purposes. B) Densitometry analysis of expression in treated versus untreated samples when normalised to *beta actin*. Data is graphed as mean ± standard error of mean. (n = 3). (DAC – 5-aza-2′deoxycitidine).

Following treatment of cells with a DNA methyltransferase inhibitor (DAC), the effects on CXC (ELR+) family expression were examined using RT-PCR (Figure 5). A significant reduction of *CXCL3* (p<0.01) was observed in the HBEC4 cell line and not in any NSCLC cell lines (Figure 5B). *CXCL8* expression was significantly induced in two of the three cell lines (HBEC4/SKMES-1 – p<0.05, Figure 5B). The two receptors *CXCR1* and *CXCR2* were significantly induced by DAC in HBEC4 (p<0.01) (Figure 5A, B). DAC demonstrated the ability to reactivate *CXCR1* but not *CXCR2* expression in SKMES-1 (p<0.01) (Figure 5A, B). DAC could induce *CXCR2* but not *CXCR1* in A549 (p<0.05) (Figure 5A, B). While this data would suggest that DNA CpG methylation is involved with the regulation of expression of the CXC receptors and CXCL8, a search of the UCSC genome library (http://genome.ucsc.edu/) revealed a sparse number of CpG residues at the promoter regions of these three genes. Therefore, we believe a change in the expression levels of this family maybe due to a secondary effect caused by DAC treatment, such as the up-regulation of specific transcription factors.

### Cellular proliferation of SKMES-1 is decreased in the presence of recombinant CXCL8, while increased in A549

**Figure 6:**
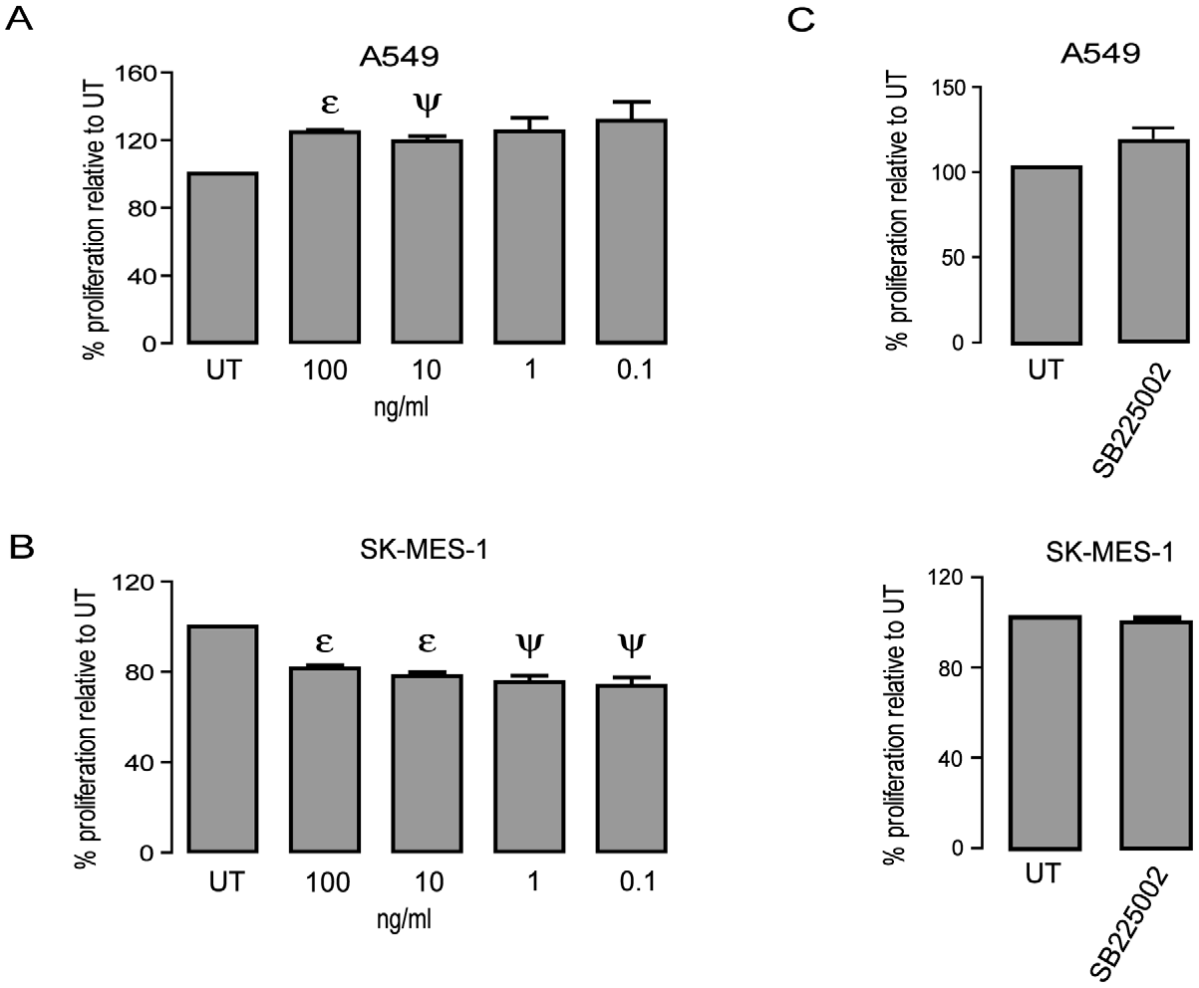
The growth effect of CXCL8 varies between NSCLC sub-type but is negated by neutralizing the CXCR2 receptor. A) Cell proliferation was examined by BrdU assay following 24–72 h treatment with CXCL8 in A549 (72 h post treatment) and SKMES-1 (24 h post treatment) cell lines. Only time points with significant data is shown. Data is represented as a percentage of the untreated control (UT), which was set to 100% and is expressed as mean ± SEM. (n = 3) (ε p<0.01 – CXCL8 treatment *vs.* UT, ψ p<0.05 **–** CXCL8 treatment *vs.* UT) B) Cell lines were treated with a selective CXCR2 antagonist (0.022µM) for 1 h prior to the addition of CXCL8 and cultured for a period of 24 (SKMES-1) or 72 h (A549). Data is represented as a percentage of the untreated control (UT), which was set to 100% and is expressed as mean ± SEM. (n = 3).

When treated with various concentrations of recombinant CXCL8 (0.1–100 ng/mL) for a period of 24–72 h, there was a trend for a decrease in proliferation in the HBEC cell line relative to untreated control (data not shown). CXCL8 treatment (100 ng/mL and 10 ng/mL) caused an increase in proliferation in the A549 cell line (p<0.01–100 ng/mL, p<0.05–10 ng/mL) at 72 h post treatment only (Figure 6A). However, there was a significant decrease in proliferation at 24 h post treatment in SKMES-1 (Figure 6B) at all concentrations tested (p<0.01, 100 ng/mL and 10 ng/mL; p<0.05 1 ng/mL and 10 ng/mL) but not at any other time point (data not shown). To determine if the proliferative effect in both of these cell lines was specifically due to CXCL8 treatment, a CXCR2 neutralising approach was undertaken. Cell lines were treated with SB 225002 at a previously published IC_50_ value of 22 nM [33] for one hour prior to the addition of CXCL8 at 100 ng/mL for 72 h (A549) and 24 h (SKMES-1) (Figure 6C). Blockade of the CXCR2 receptor negated the proliferative effect in both A549 and SKMES-1 cell lines compared to CXCL8 treatment alone.

## Discussion

The CXC (ELR^+^) chemokine family, a potent pro-angiogenic family, was found to be regulated epigenetically in both NSCLC and normal bronchial epithelial cell lines at the level of both histone post-translational modifications.

In a panel of normal/tumour matched samples, the chemokines and receptors displayed no altered expression pattern between adenocarcinoma and squamous cell carcinoma samples, with the exception of *CXCL3* (average value elevated in squamous cell carcinoma and reduced in adenocarcinoma). Higher levels of CXCL8 have been detected by IHC in lung cancer tissue samples when compared with normal [34], and in our samples levels of *CXCL8* mRNA were elevated in 37.8% (14/37) samples. Although the overall average densitometry values (adenocarcinoma n = 14 and squamous cell carcinoma n = 23) indicate a reduction of chemokines in the tumour samples (Figure 1C), this was not true for every individual sample (Figure 1 A). Due to the heterogenicity of the samples, it is difficult to make a definitive conclusion based on these results. It must be noted however, that expression of many of the chemokines were most often found to be downregulated in the NSCLC tumours as follows *CXCL1* (64.9%, p<0.01), *CXCL2* (81.1%, p<0.01), *CXCL3* (59.5%) (Figure 1B). In addition, levels of *CXCR1* were also found to be significantly reduced in NSCLC tumours (35.2%, p<0.05).

In a panel of NSCLC cell lines treated with HDACi there was a decrease in *CXCL1–3* with a concomitant increase in *CXCL8* and the receptors *CXCR1* and *2* (Figure 3B). TSA has previously been shown to up-regulate CXCL8 in lung [35] and breast cancer cell lines [36]. The reactivation of these receptors in the lung cancer cell lines would indicate that they are under epigenetic regulation at the level of histone modification. The general dogma associated with HDACi is that they function to induce gene expression. However in this study, TSA caused both an up- and down- regulation of the chemokines, an effect seen by others in other studies. For example, HDACi have been shown to down-regulate Wilms tumour gene 1 (Wt1) [37] and EGFR [38]. Limited availability of specific transcription factors may result in only a certain number of up-regulated genes. As such the activation of *CXCL8* may lead to the down regulation to *CXCL1–3*, either via a molecular switch or through the titration of transcription factors away from the promoter regions of these genes. ChIP results indicate that TSA acts by directly remodelling the promoter region of the *CXCL8* and *CXCR1/2* genes (Figure 4). Our ChIP results show that histones H3 and H4 become hyperacetylated at these gene promoters, involving lysines 9 and 14 of histone H3, and lysines 5, 8, 12 and 16 of histone H4. We observe a strong increase in the levels of histone H3 lysine 4 dimethylation (H3K4me2) following activation of transcription via HDACi, and also observe a loss of histone lysine 4 monomethylation (H3K4me), following activation of CXCL8/CXCR1/CXCR2. In agreement with the current literature, we observe the presence of a repressive mark H3K9Me2 at the PGIS promoter prior to activation via HDACi, and levels of this histone modification decrease following activation [39], [40]. These results verify that HDACi causes causes chromatin remodelling via histone post-translational modifications around the promoter region of the genes examined indicating that it is active element in the regulation of these chemokines and their receptors.

DNA CpG methylation is also a major component in epigenetic regulation of gene expression. Hypermethylated DNA is often found in the promoter regions of tumour suppressor genes in cancers, particularly in lung cancer [30]. Treatments with the DNMTi DAC reactivated the expression of *CXCR1* in the A549 (Figure 5B) cell line and *CXCR2* in SKMES-1 (Figure 5B). However, searches of both promoter databases and the genome failed to find any significant CpG islands in the promoter regions of these genes, indicating therefore that the reactivation of these receptors is an indirect effect of DNMTi treatment, perhaps by the reactivation of a transcription factor necessary for these genes to be transcribed.

The CXC (ELR^+^) chemokines are important in neo-vascularisation and metastasis. This study has demonstrated that CXCL8 can be up-regulated by epigenetic targeting using HDACi or DNMTi. Current literature indicates that CXCL8 is a chemokine involved in carcinogenic processes by stimulating cellular proliferation in some NSCLC cell lines [41], [42], [43], but results are conflicting [44]. In this study, treatment with recombinant CXCL8 failed to induce proliferation in a normal cell line (data not shown) and significantly decreased proliferation in the SKMES-1 cell line at 24 h post treatment (Figure 6B). Conversely proliferation was increased in A549 after 72 h exposure to CXCL8 (Figure 6A). Treatment with a selective non peptide CXCR2 inhibitor (SB 225002) prevented the proliferative alterations attributable to CXCL8 treatment (Figure 6C), indicating that such responses were due to CXCL8 signalling through functional CXCR2 receptors.

Very low basal receptor expression was observed in the NSCLC cancer cells, whereas expression was strong in the normal cell lines (Figure 2), which may suggest that the receptors are important in normal physiological processes. Although the expression of CXC receptors correlates with increased tumour angiogenesis and growth, in some circumstances the loss of receptor expression may contribute to carcinogenesis [8]. A recent study published by Ohri *et al*, found that a high expression of CXCR2 in tumour islets is associated with increased survival in NSCLC [45]. In human fibroblasts, CXCR2 has been shown as an important mediator of senescence, activating a DNA damage check-point [46]. Therefore, the loss of CXC receptor expression may allow for neoplastic cells to escape senescence, resulting in cancer promotion. The seemingly dual function of CXCR2 may explain the conflicting results concerning CXCL8 as a growth factor. Other findings suggest that CXCL1 or CXCL8 signalling may reduce tumour growth by promoting senescence through CXCR2 [47]. Therefore, epigenetic targeting of this family may be of potential therapeutic benefit, as our results indicate that CXCR2 and CXCL8 can be induced by epigenetic means.

Chemokines are important in cancer as they possess the ability to promote organ specific metastases making them an attractive therapeutic target, particularly as 90% of cancer related deaths are due to tumour metastases. Our results clearly demonstrate that the expression of the CXC (ELR^+^) family is commonly disrupted in NSCLC. Furthermore, we have established that these chemokines and their cognate receptors are regulated epigenetically, and may represent good targets for epigenetic therapy in the treatment of NSCLC. Further experimental work will be necessary to delineate these possibilities and to determine if the aberrant epigenetic regulation of these genes plays a role in NSCLC pathogenesis.
